# Primary giant mediastinal synovial sarcoma of the neck: A case report and review of the literature

**DOI:** 10.3892/ol.2013.1649

**Published:** 2013-10-29

**Authors:** YAN ZHOU, WEN DONG, FANGWEN ZOU, DONG-AI ZHOU, JIN-AN MA

**Affiliations:** Department of Oncology, The Second Xiangya Hospital of Central South University, Changsha, Hunan 410011, P.R. China

**Keywords:** synovial sarcoma, mediastinum, neck mass

## Abstract

Synovial sarcomas commonly occur in the soft tissue of the extremities, while a primary occurrence in the mediastinum is quite rare. The current study reports the case of an 11-year-old male who presented with a neck mass, which computed tomography showed was due to a giant mediastinal mass involving the thyroid gland. The tumor was resected by thoracotomy and diagnosed as monophasic synovial sarcoma by histopathology. The patient received adjuvant combination chemotherapy and radiation therapy following surgery. At the 3-month follow-up, no local tumor recurrence was found. The present case report highlights the significance of recognizing the unusual presentation and clinical manifestation of synovial sarcoma to aid clinical management. Written informed consent was obtained from the patient’s family.

## Introduction

Synovial sarcoma is a type of mesenchymal tissue cell tumor that exhibits epithelial differentiation, which most frequently arises in the extremities, while a primary occurrence in the mediastinum is quite rare (1,2). Primary mediastinal synovial sarcomas are malignant tumors with a low incidence, no specific clinical manifestations and a lack of unified and effective treatments, highlighting the challenges preventing its diagnosis and treatment in clinics. The present study reports a case of primary giant mediastinal synovial sarcoma of the neck in a patient admitted to The Second Xiangya Hospital of Central South University (Hunan, China).

## Case report

**Figure 1:**
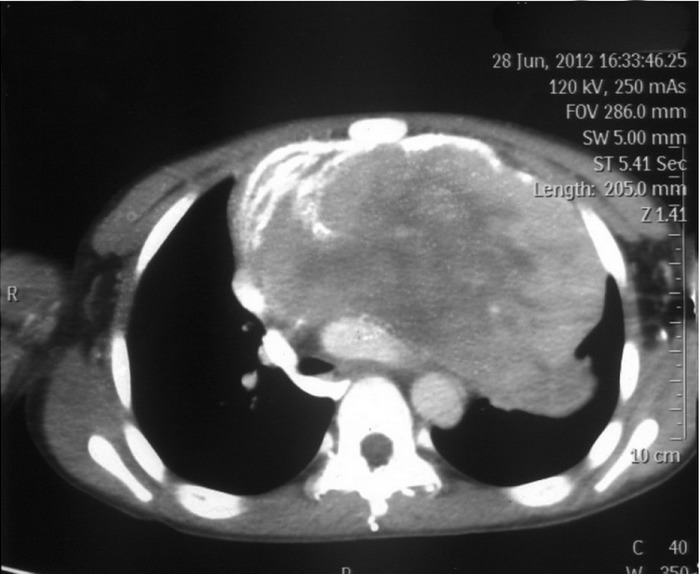
CT scan showing a large mass, 14.1×6.7 cm^2^ in size, in the anterior mediastinum. CT, computed tomography.

**Figure 2:**
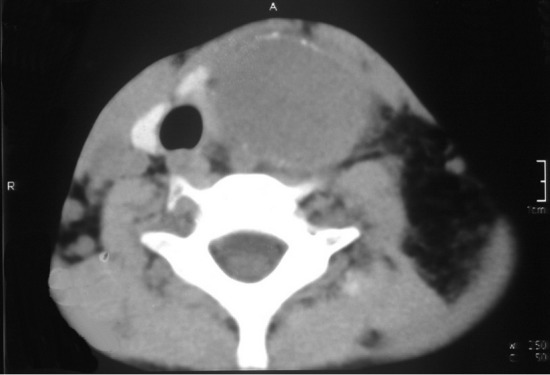
CT scan showing that the mass extends into the left thyroid gland. CT, computed tomography.

**Figure 3:**
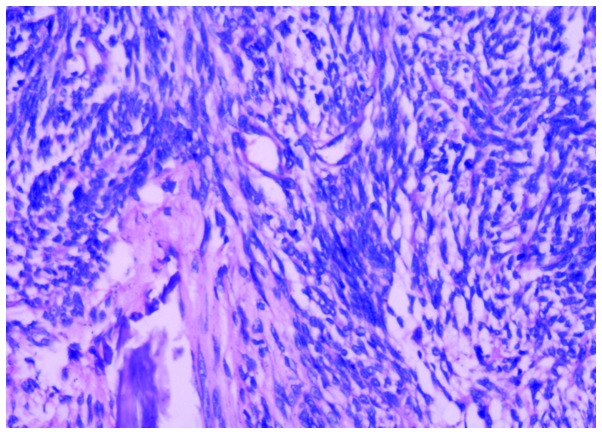
HE staining showing malignant mesenchymal tumor tissue. HE, hematoxylin-eosin.

**Figure 4:**
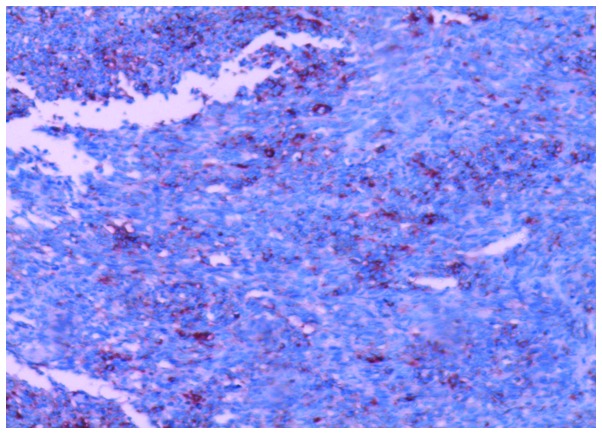
Immunochemistry examination revealing EMA(+) tissue.

**Figure 5:**
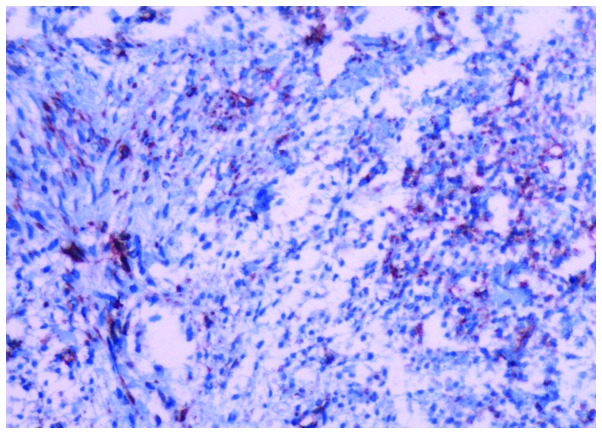
Immunochemistry examination revealing VIM(+) tissue.

**Figure 6:**
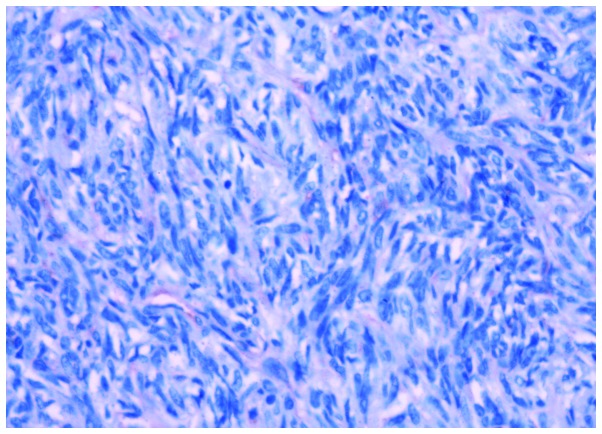
Immunochemistry examination revealing CD99(+) tissue.

**Figure 7:**
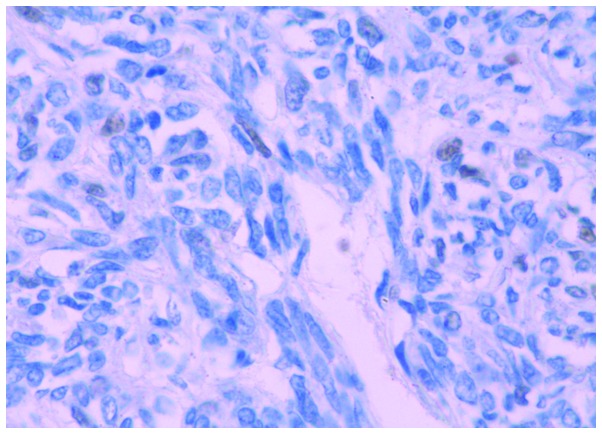
Immunochemistry examination revealing S100(+) tissue.

**Figure 8:**
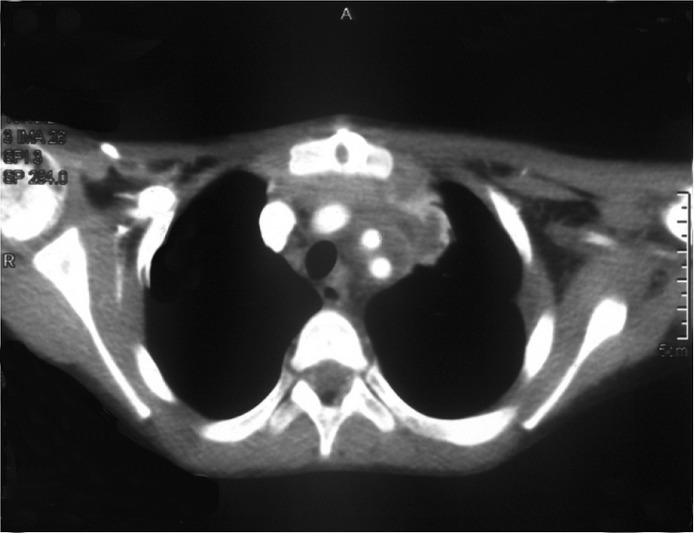
Follow-up CT subsequent to surgery showing a soft tissue mass of 5 cm in diameter found in the anterior mediastinum. CT, computed tomography.

**Figure 9:**
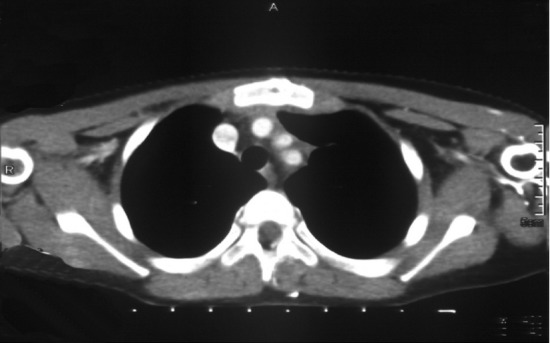
Follow-up CT subsequent to chemotherapy revealing no mass in the mediastinum. CT, computed tomography.

An 11-year-old male presented with a mass in the left side of the neck that had been present for ~1 month, together with mild dysphagia, without coughing, chest pain or shortness of breath. A physical examination revealed a large lump with an area of ~4×3 cm^2^ in the left thyroid gland, however nothing of significance was revealed in the chest or abdominal regions. Computed tomography (CT) scans (Figs. 1 and 2) showed a large, patchily enhanced mass in the anterior mediastinum, which extended into the left thyroid gland. Patchy areas of necrosis, low-density liquidity and calcification were observed in the mass. A thoracotomy was performed, revealing a huge mass measuring 20×15×15 cm^3^, arising from the anterior mediastinum, violating the lower pole of the thyroid and engulfing the left phrenic nerve and innominate vein. The left innominate vein and phrenic and vagus nerves were resected and bluntly separated, and then the mass was removed intact whilst the left diaphragmatic muscle was simultaneously suspended. A histological examination (Fig. 3) showed malignant mesenchymal tumor tissue involving the left innominate vein. An immunochemistry examination revealed the following results: EMA(+), Vim(+), S100(+), CD99(+), Ki-67(+), CD117(−), CD34(−), SAM(−), HMB(−), CK(−) and p53(−) (Figs. 4–7), confirming the lesion as a monophasic synovial sarcoma. One month after surgery, a follow-up CT scan (Fig. 8) showed a patchily enhanced soft tissue mass, 5 cm in diameter, in the anterior mediastinum, while the neck structure was clear. Adjuvant chemotherapy containing ifosfamide, 1.8 g days 1–4 and doxorubicin, 30 mg days 1–2, administered every 3 weeks for four cycles, was proposed for the patient due to the residual mass. A thoracic CT (Fig. 9) was performed again following chemotherapy and revealed no mass in the mediastinum or neck. A total of 64 Gy adjuvant radiotherapy was applied to the primary tumor with hope of an increased disease-free survival. The patient recently completed treatment and is currently undergoing follow-up.

## Discussion

Synovial sarcoma was defined by the World Health Organization (WHO) in 2002 as a type of mesenchymal tissue cell tumor that exhibits epithelial differentiation (1), which most frequently arises in the extremities and has been prevalent in adolescent and young adults between the ages of 15–40 years (2). Although 85% of synovial sarcomas arise in joint cavities, they may also occur in locations unassociated with joint cavities, including the head and neck, thoracic wall, abdominal region, genitourinary tract and other rarer sites (3). In a retrospective study conducted by Burt *et al*(4), of the total 3,149 soft tissue sarcomas examined, primary mediastinal sarcoma represented 1.4%. The most common primary mediastinal sarcoma was the malignant peripheral nerve sheath tumor, which accounted for 26%, while the synovial sarcoma only accounted for 2%. To date, only 24 previous studies analyzing mediastinal synovial sarcoma have been found through searching PubMed, in which patients’ ages ranged between 3 and 83 years, with a male-to-female ratio of ~3:1 and tumors measuring between 5 and 20 cm in their greatest diameter. Histologically, 36 cases exhibited a biphasic growth pattern and monophasic type at a ratio of 1:1 and 1 case exhibited a poorly-differentiated form. The overall survival time ranged between 3 months and >5 years, with a median overall survival time of 19.8 months.

Primary mediastinal synovial sarcoma is a type of malignant tumor with no specific differences from other mediastinal tumors with regard to clinical manifestation, imaging or histology, therefore, it is difficult to diagnose. With regard to clinical manifestations, mediastinal synovial sarcomas reveal various initial symptoms due to their different scopes of infringement. The common symptoms include chest pain (5–7), shortness of breath and dyspnea (8,9), in contrast to the present case whose initial symptom was the accidental identification of a neck lump, which is a rare clinical manifestation and is likely to be easily ignored and misdiagnosed. Therefore, it is important to combine imaging and pathology to assist in establishing a diagnosis. With regard to imaging performances, the majority of mediastinal synovial sarcoma patients visit their doctors following the onset of dyspnea or other manifestations. Chest X-rays or CT scans may reveal space-occupying lesions in the mediastinum, which exhibit no specific radiological characteristics to other mediastinal stromal tumors, including necrotic, hemorrhagic or cystic components on section, and calcification may be found in the mass. In addition, magnetic resonance imaging or positron emission tomography/CT may demonstrate the adhesion and invasion scope of lesions to the surrounding tissue, thus offering guidance for the selection of appropriate treatment. The pathological diagnosis is important since the multiformity of synovial sarcoma in clinical manifestations and the absence of specificity relative to imaging performances have limitations for the diagnosis of synovial sarcoma. Usually, patients are confirmed to have synovial sarcoma by B ultrasound or CT-guided fine-needle aspiration cytology from the mass, or by post-operative pathological examination. Pathological diagnosis remains the gold standard, and synovial sarcoma is divided into four types according to the various histological observations of epithelial and spindle cells in the mass (10), including monophasic spindle and epithelial cell types, a poorly-differentiated form and a biphasic pattern. In addition, characteristic histopathological observations and immunochemistry examinations are important for the differential diagnosis of a synovial sarcoma from other stromal tumors (11). It has been indicated that vimentin, cytokeratin and EMA positivity, in combination with CD34 negativity, are the most useful protein biomarkers for the diagnosis of monophasic synovial sarcoma (10). In the current case report, the tumor was confirmed as a malignant mesenchymal tumor under the microscope, but its subtype was difficult to determine. The tumor cells were found to be positive for vimentin, EMA and S100, and negative for CD34, which, in combination with the morphological profile, confirmed the diagnosis of a monophasic synovial sarcoma. With regard to a genetic diagnosis, cellular and molecular genetic studies (10–13) have shown that the translocation t(X;18)(p11.2; q11.2) exists in >90% of synovial sarcomas. The translocation involves the SYT gene on chromosome 18 and the SSX1 or SSX2 gene on the X chromosome. In addition, this translocation is not associated with other sarcomas and thus, may represent a specific biomarker for diagnosing synovial sarcoma. However, this fusion gene was not detected in the present case.

Synovial sarcomas, particularly mediastinal synovial sarcomas, are highly aggressive tumors, which are more likely to invade adjacent significant organs, including the heart, lung and blood vessels, the majority of which exhibit no evident clinical features, thus highlighting challenges for diagnosis and prompt treatment. The following treatments have been applied for synovial sarcomas: i) Although mediastinal synovial sarcomas have limited clinical data and no standardized treatments, complete surgical excision remains the cornerstone of therapy (5,6,8,9). A broad surgical excision must be promoted for inchoate patients, as presented in the current case report where the patient’s tumor and left innominate vein and phrenic nerve were resected in hope of increased disease-free survival. For patients with a neoplasm that has invaded into the adjoining organs, the partial excision method may provide appropriate clinical management. Ferrari *et al*(14) retrospectively studied 271 patients with synovial sarcomas and found that the 5-year disease-free survival rate was 42.5% for patients treated with complete surgical excision and 31.6% for patients treated with partial surgical excision. The remaining 31.9% of patients were not treated with surgery. A partial surgical excision may not prolong disease-free survival, as indicated by these results, but it may solve the issues of obstruction or filling for terminal patients who may not be treated by surgical excision due to a wide range of violations, thus, there remains specific implications. ii) Radiotherapy is an effective treatment method to kill cancer cells and control local recurrence rates. In the retrospective study conducted by Ferrari *et al*(14), adjuvant radiotherapy did not prolong the 5-year local recurrence-free survival (LRFS) rate in patients with completely resected disease and free histological margins; the 5-year LRFS rate for patients who received post-operative radiotherapy and for those who had not was 77.8 and 66.9%, respectively. However, adjuvant radiotherapy showed a marked impact on patients with marginally resected disease, as in the 71 patients who received partial surgical excision, the 5-year LRFS rate was 57.4% for patients who received radiotherapy and only 7.1% for patients who had not. Similarly, Harb *et al*(15) also found that patients with synovial sarcoma of the head and neck who were treated with surgery and post-operative radiotherapy exhibited higher survival and lower recurrence rates than those treated with surgery only or a combination of surgery and chemotherapy. There has been a lack of reliable data analysis to determine the significance of post-operative radiotherapy in mediastinal synovial sarcoma, but according to the aforementioned data, mediastinal synovial sarcoma patients are commonly treated with adjuvant radiotherapy following surgery, particularly those patients with positive histological margins. In the current case report, a large tumor was found in the mediastinum prior to surgery, and a small mass shadow remained visible in the follow-up CT scan following surgery. After referring to specific mediastinal synovial sarcoma treatments available overseas (6,9), the patient was treated with adjuvant post-operative radiotherapy with a total of 64 Gy, in the hope of controlling local recurrence. iii) Chemotherapy is an additional, predominant tool used for tumor treatments and is particularly important for preventing tumors from distant metastasis. Synovial sarcomas have moderate chemosensitivity with a response rate of 50% to regimens containing ifosfamide and doxorubicin (16). In the study by Ferrari *et al*(14), the 5-year metastasis-free survival rates were 60.2% [standard error of the mean (SEM), 6.8%] and 47.8% (SEM, 3.3%) for patients who had accepted adjuvant chemotherapy and for those who had not, respectively. Further analysis has shown that the greatest benefits associated with chemotherapy usually appear in patients ≥17 years old, with tumors measuring >5 cm. In addition, neoadjuvant chemotherapy is likely to reduce tumor size and thus, may offer surgical conditions for patients who cannot undergo surgery immediately, and therefore improve radical surgical resection rates. Balieiro *et al*(17) recently reported a case of a giant primary mediastinal synovial sarcoma treated with neoadjuvant chemotherapy. A large mass of 20 cm in its largest diameter was found in the upper section of the mediastinum and had invaded the main anterior vessels and chest wall, and compressed the heart and critical left mainstem bronchus, thus, the patient exhibited a lack of surgical indications. The mass was significantly reduced following 6 cycles of neoadjuvant chemotherapy (ifosfamide and doxorubicin) followed by radical en bloc resection, and the patient exhibited no signs of disease recurrence following five years of routine follow-up examinations. The therapeutic regimen of ifosfamide and doxorubicin has been regularly used for the treatment of synovial sarcomas to prolong overall survival rates (6,7,9,17). The patient in the present case report also accepted 4 cycles of this combination regimen and the follow-up CT scans (Fig. 8 and 9) showed that the mass in the mediastinum had shrunk, confirming that the chemotherapy had worked. iv) The final treatment for synovial sarcoma is targeted therapy. In total, >90% of synovial sarcoma patients have the aforementioned fusion genes, SYT-SSX1 or SYT-SSX2. In a study conducted by Sarver *et al*(18), it was revealed that the SYT-SSX gene may target EGR1 receptors to inhibit the expression of EGR1, a type of cancer suppressor gene, and thus, is involved in cell migration. Studies with regard to the role of this highly-expressed fusion gene in synovial sarcoma (19) must be investigated in hope of identifying novel targeted therapy drugs.

A previous study (3) showed that the 5-, 10- and 15-year survival rates of synovial sarcoma patients who received complete surgical excision and adjuvant radiotherapy were 76, 63 and 57%, respectively. However, synovial sarcomas in the mediastinum have a poorer prognosis compared with synovial sarcomas presenting in the extremities, since synovial sarcoma may be more likely to invade the surrounding organs, including the heart, lungs and blood vessels. The existing clinical data have shown that the overall survival time for mediastinal synovial sarcoma is between 3 months and >5 years (7–9,17). Poor prognostic factors may include being of the male gender, being >20 years old, tumor diameters of ≥9 cm, the existence of extensive tumor necrosis, neurovascular invasion and the presence of the SYT-SSX1 or SYT-SSX2 fusion genes (9).

Mediastinal synovial sarcomas are malignant tumors with a low incidence, no specific clinical manifestations and a lack of unified and effective treatments. These factors highlight the challenges preventing its diagnosis and treatment in clinics. The existing data supports surgery as the preferred treatment for mediastinal synovial sarcoma, but appropriate auxiliary treatments, including radiotherapy and chemotherapy, must be taken into consideration according to the factors affecting prognosis. In addition, the SYT-SSX fusion gene in synovial sarcoma must be further investigated in hope of identifying novel targeted therapies.
